# Effects of renal denervation on endogenous ouabain in spontaneously hypertensive rats

**DOI:** 10.1590/acb371102

**Published:** 2023-01-06

**Authors:** Xiaomei Lai, Hong Wen, Tingting Yang, Fei Qin, Xiaoge Zhong, Yajin Pan, Jie Yu, Jing Huang, Jianling Li

**Affiliations:** 1 Postgraduate. Guangxi Medical University – First Affiliated Hospital – Department of Cardiology – Nanning, China.; 2 PhD. Guangxi Medical University – First Affiliated Hospital – Department of Cardiology – Nanning, China.; 3 PhD, and Postdoctoral Mobile Station. Guangxi Medical University – First Affiliated Hospital – Department of Cardiology – Nanning, China.

**Keywords:** Ouabain, Hypertension, Sodium-Potassium-Exchanging ATPase, Rats

## Abstract

**Purpose:**

To investigate the role of renal denervation (RDN) on endogenous ouabain (EO) secretion in spontaneously hypertensive rats (SHR).

**Methods:**

Sixteen 12-week-old male SHR were randomly separated into the renal denervation group (RDNX group) and sham operation group (sham group), and eight age-matched Wistar Kyoto rats (WKY) were served as control group. EO concentrations, the Na^+^- K^+^-ATPaseactivity, and the expression of Na^+^-K^+^-ATPase were assessed.

**Results:**

EO levels in serum, kidneys and hypothalamus of sham group were higher than in RDNX group (p < 0.05). Renal Na^+^-K^+^-ATPase activity subjected to denervation surgery showed significantly reduction when compared with the sham groups (p < 0.05). A positive correlation existed between norepinephrine (NE) content and Na^+^-K^+^-ATPase activity in the kidney (r^2^ = 0.579). Renal Na^+^-K^+^-ATPase α1 subunit mRNA expression was down-regulated in the RDNX group compared with the sham group (P < 0.05), while renal Na^+^-K^+^-ATPase α1 subunit mRNA expression was no statistical significance between the groups (P = 0.63). Immunohistochemical analysis showed that there were significant differences in the renal expression of Na^+^-K^+^-ATPasebetween the three groups (P < 0.05).

**Conclusions:**

These experiments demonstrate that RDN exerted an anti-hypertensive effect with reduction of EO levels and Na^+^-K^+^-ATPase activity and Na^+^-K^+^-ATPase α1 subunit expression of kidney in SHR.

## Introduction

Endogenous ouabain (EO) was first identified in human plasma in 1991^1^, which was an endogenous Na^+^-K^+^-ATPase inhibitor^2^. Hamlyn *et al*.^2^^,^ 3 found that EO in blood of animals with increased blood volume can regulate the Na^+^-K^+^-ATPase activity, promote the excretion of sodium from the kidneys and cross-react with digoxin antibody. EO also demonstrated the cardiotonic, diuretic, vasoconstrictive, and other digitalis-like effects. The vascular effect of EO includes inhibition of the Na^+^-K^+^-ATPase α2 subunit that increases intracellular Na^+^, which promotes Ca^2+^ entry into the intracellular by reducing the activity of the sarcolemmal Na^+^/Ca^2+^ exchanger, and thereby increases the vascular smooth muscle tone^4^. On the other hand, the renal effect involves the ability of EO to stimulate Na^+^ transport across the tubular cell by activating the basolateral Na^+^-K^+^-ATPase α1 subunit, ultimately contributing to increase blood pressure (BP)^5^.

Disorders of water and sodium metabolism are the pathophysiological basis of essential hypertension^6^. The kidney plays a primary role in the regulation of sodium and potassium homeostasis^7^. Furthermore, the change of EO content in the circulation corresponds to the status of sodium balance^8^. However, the cause of hypertension induced by EO remains poorly understood. However, it is generally believed that EO may combine with Na^+^-K^+^-ATPase in cell membrane, which results in altered gene expression of Na^+^-K^+^-ATPase α-subunit and enzyme conformation responsible for its activities^9^. Consequently, it’s suggesting that a possible association between ouabain and the genesis or development and maintenance of hypertension and, moreover, ouabain may be a related factor of essential and secondary hypertension^8^.

Sympathetic nerve fibers innervate all organs that are involved in cardiovascular control, such as the heart, peripheral blood vessels and, perhaps most importantly in the current context, the kidneys. The sophisticated network of efferent sympathetic and afferent sensory nerve fibers residing in the kidney and their signaling pathways provide the basis for the modulating influence of central integrative structures in the brain stem on renal effectors contributing to BP control^10^^,^ 11. Increased renal sympathetic nerve activity promotes the development of hypertension through the activation of the renin-angiotensin-aldosterone system (RAAS)^12^^,^ 13. Based on all these mechanisms, recently, several studies indicate that bilateral renal denervation (RDN) has emerged as safe and effective, and either prevented, delayed the onset, or reduced the magnitude of the hypertension^14^^-^ 16.

The clinical implication and perspectives of RDN-based hypertension management strategy have caught the attention of many scientific groups. Animal studies pointed to increase EO content triggering sympathetic nerve activity elevation and evoking hypertension^17^. Angiotensin II (Ang II)-, aldosterone-, or salt and renal injury-induced forms of hypertension can be effectively controlled by blockade of any step in a neuromodulatory pathway of local EO^18^. However, the effect of RDN on EO secretion in spontaneously hypertensive rats remains poorly understood. Hence, the aims of this study were to investigate the effects of RDN on the expression of Na^+^-K^+^-ATPase α1 and β1 subunits in spontaneously hypertensive rats (SHR), and further to explore the role of renal sodium handing treatment by RDN.

## Methods

### Animals and bilateral renal denervation

All procedures were performed following the Animal Ethics Committee guidelines of Guangxi Medical University and had approval. Ten-week-old male spontaneously hypertensive rats (SHR group, n = 16) and age-matched Wistar-Kyoto rats (WKY group, n = 8), normotensive controls, were purchased from Vital River Laboratories Co., Ltd. (Beijing, China). Specifically, after two weeks of adaptive feeding, the SHR were randomly divided into two groups, the renal denervated group (RDNX, n = 8) and sham operation group (sham group, n = 8). Animals were maintained in standard laboratory animal housing conditions.

After sterilization, a midline incision was made. Then, bilateral RDN was induced by painting the renal vessels with 10% phenol under 2% pentobarbital sodium anesthesia [50 mg/kg, i.p.] in RDNX group. While the sham group and the WKY group underwent the same procedure as the RDNX group, the renal vessels were painted with normal saline. Two weeks after surgery, under ketamine anesthesia [200 mg/kg, i.m.], blood was collected via the abdominal aorta, and the kidneys, adrenal glands and hypothalamus were removed and stored at -80 °C until analysis. Furthermore, the effectiveness of RDN had already been testified in a previous examination from our laboratory^19^.

### Determination of endogenous ouabain

After blood collection from the abdominal aorta, the blood samples were allowed to stand at room temperature for 30 min. Then, the serum was separated at 1,500 rpm for 10 min. Tissue samples were thawed and minced, and 2% homogenate was made by adding appropriate amount of saline on ice and centrifuged at 2,500 rpm for 10 min to extract the supernatant. The concentrations of EO in serum and tissue were determined using the radioimmunoassay method with an ^125^I RIA kit. The intra- and inter-assay coefficients of variation, respectively, were less than 10 and 15% for EO.

### Na^+^-K^+^-ATPase activity assay

The kidneys were thawed, weighed, and homogenized in ice-cold saline to 10%, and the homogenates were centrifuged at 2,500 rpm for 10 min at 4 °C. Then, 200 μL of supernatant was extracted and diluted into 2% tissue homogenate by adding 800 μL of saline. The concentration of proteins was measured by Coomassie brilliant blue method. The activity of Na^+^-K^+^-ATPasewas determined in the supernatant spectrophotometrically with the corresponding kit (Nanjing Jiancheng Biochemistry Co., Nanjing, China). Enzyme activity was expressed as micromoles of Pi released per hour per milligram of protein.

### Real-time polymerase chain reaction

RNA was extracted from kidneys using RNAsimple Total RNA Kit (Tiangen Biotech). Total RNA was reverse transcribed using a cDNA Synthesis Kit (Takara) to acquire cDNA. The sequences for the specific primers used are shown in Table 1. Real-time polymerase chain reaction (PCR) was conducted using a Realplex4 Mastercycler Real Time PCR System (Eppendorf) and a SYBR^®^ Premix Ex Taq™ Kit (TaKaRa), under universal cycling conditions (95 °C for 30 sec, 95 °C for 5 sec, and 60 °C for 30 sec, 40 cycles). The products were confirmed by appropriate size by agarose gel electrophoresis. The samples were assayed in triplicate. Glyceraldehyde-3-phosphate dehydrogenase (GAPDH) was used as endogenous control to normalize the transcript levels, and data were analyzed according to the comparative threshold cycle method.

**Table 1:** Primer sequences used for quantitative real-time polymerase chain reaction.

Gene	Primer sequences (5’-3’)		Amplicon size (bp)
Na^+^-K^+^-ATPase α1	F	CTGATCAGCATGGCCTATGGAC	87
	R	ACCGTTCTCAGCCAGAATCACA	
Na^+^-K^+^-ATPase β1	F	CTGCTTACCATCAGTGAGCTGAAAC	136
	R	GCACATAGGCCTCGTAGCTCTTG	
GAPDH	F	GGCACAGTCAAGGCTGAGAATG	143
	R	ATGGTGGTGAAGACGCCAGTA	

F: forward; R: reverse; bp: base-pairs; GAPDH: glyceraldehyde-3-phosphate dehydrogenase.

### Immunohistochemistry

In brief, kidney slices (3 μm) were deparaffinized in xylene and dehydrated in a graded series of ethanol after formalin fixation and paraffin embedding. The sections were incubated in 3% H_2_O_2_ for 5 min to suppress endogenous peroxidase activity after heating in citrate buffer sodium (pH 6) and blocked with 10% normal goat serum for 10 min to prevent nonspecific binding. Next, the slides were incubated with anti-Na^+^-K^+^-ATPase antibody for 3 h at room temperature in a humidified chamber. All sections were incubated with SP-9000 reagent for 20 min at 37 °C. Immunoreactivity was observed with 3,3’diaminobenzidine reagent and applied with Harris hematoxylin to counterstain. All kidney sections were washed, dried, and mounted. Images were acquired using Digital Microscope Imaging System (DP70, Olympus, Tokyo Japan) and expressed by mean density using computer-assisted image analyzer system (IPP 6).

### Statistical analysis

Results are provided as means ± standard error and processed with SPSS 16.0 statistical software (SPSS Inc. Chicago, IL, United States of America). Differences were determined using analysis of variance (ANOVA) when more than two groups were compared followed by the Student-Newman-Keuls-q test or Dunnett-t’s test. Pearson correlation analysis was used to evaluate the relationship between variables. Significance was declared at p < 0. 05.

## Results

### Effect of renal denervation on serum and tissue endogenous ouabain levels

**Figure 1:**
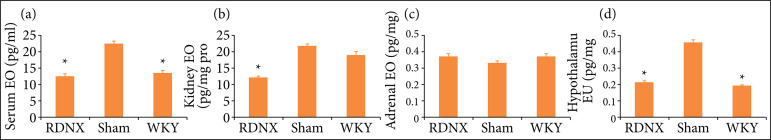
EO content in different parts in different groups: **(a)** serum from abdominal aorta; **(b)** kidney; **(c)** adrenal gland; **(d)** hypothalamus.

EO: endogenous ouabain; RDNX: renal denervation group; WKY: Wistar Kyoto rats. Significances (P < 0.05) are * vs. sham.

As shown in Fig. 1, renal denervation significantly decreased EO levels of serum as compared to sham group with intact renal innervation (12.32 ± 3.02 vs. 22.04 ± 2.68, P < 0.05). The kidneys and hypothalamus of EO concentrations were significantly lower in RDNX group compared with the sham group (P < 0.05), and there was no statistical difference between the RDNX and WKY groups. Interestingly, no significant difference in EO content of adrenal was observed among all the groups (P = 0.90).

### Effect of renal denervation on renal tissue Na^+^-K^+^-ATPase activity

**Figure 2:**
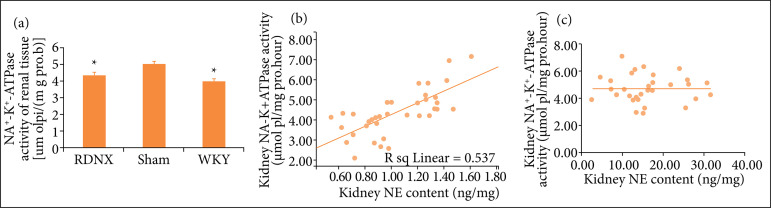
Na^+^-K^+^-ATPase activity in the kidneys: **(a)** Na^+^-K^+^-ATPase activity in different groups; **(b)** relationship between NE content and Na^+^-K^+^-ATPase activity in kidneys; **(c)** relationship between EO levels and Na^+^-K^+^-ATPase activity in kidneys.

EO: endogenous ouabain; NE: norepinephrine; RDNX: renal denervation group; WKY: Wistar Kyoto rats. Significances (P < 0.05) are * vs. Sham.

As shown in Fig. 2, Na^+^-K^+^-ATPase activity of renal tissue homogenate was significantly increased in the sham group compared with the WKY group (3.95 ± 0.97 vs. 5.02 ± 0.86 μmolpi/mgPro/hour, P = 0.02). Na^+^-K^+^-ATPase activity was slightly higher in RDNX group than in WKY group, but the difference was not significant (p > 0.05). Results of the above experiments indicated that RDN significantly decreased Na^+^-K^+^-ATPase activity of renal tissue. Data on norepinephrine (NE) renal tissue content was as previously reported^19^. Correlation analysis showed that NE content was positively correlated with Na^+^-K^+^-ATPase activity of kidney (r^2^ = 0.579, β = 0.733, p < 0.001), as shown in Fig. 2, which implied that the increased Na^+^-K^+^-ATPase activity in kidney may have been upregulated by NE. However, the correlation analysis showed no statistical difference between EO levels and Na^+^-K^+^-ATPase activity in the kidney (P = 0.93).

### Expression of Na^+^-K^+^-ATPase α1 and β1 subunits mRNA in the kidney of spontaneously hypertensive rats with and without renal denervation

**Figure 3:**
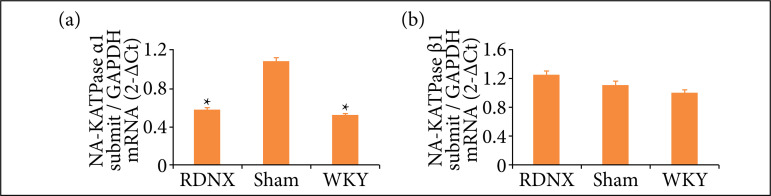
Na^+^-K^+^-ATPase α1 and β1 subunits mRNA expressions in the kidneys:
**(a)** Na^+^-K^+^-ATPase α1 subunit mRNA expressions in different groups;
**(b)**Na^+^-K^+^-ATPase β1 subunit mRNA expressions in different groups.

GAPDH: glyceraldehyde-3-phosphate dehydrogenase; RDNX: renal denervation group; WKY: Wistar Kyoto rats. Significances (P < 0.05) are * vs. Sham.

As shown in Figure 3, there was visible difference between the experimental groups in the levels of mRNA of Na^+^-K^+^-ATPaseα1 subunit in the renal tissue (0.69 ± 0.23 vs. 1.28 ± 0.29 vs. 0.62 ± 0.16, respectively, P = 0.003). In addition, the mRNA of Na^+^-K^+^-ATPase α1 subunit was markedly downregulated under chemical sympathectomy conditions compared to sham group with intact renal innervation (P < 0.05). A similar decrease was detected in the WKY group (P < 0.05). However, there was no significant difference in mRNA of Na^+^-K^+^-ATPase β1 subunit between groups (P = 0.63).

### Expression of Na^+^-K^+^-ATPase proteins in the kidney of spontaneously hypertensive rats with and without renal denervation

**Figure 4:**
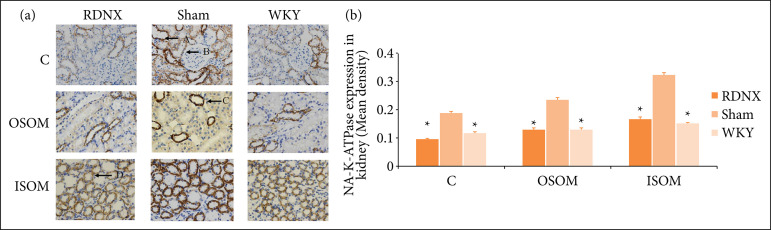
Na^+^-K^+^-ATPase immunoreactivity in different parts. **(a)** The slides are shown at 40× magnification. The expression of Na^+^-K^+^-ATPase was visible in epithelial cells of cortical distal convoluted tubules (A, *arrow*) and straight distal tubules (C, *arrow*), also detected in some of proximal tubule (B, *arrow*) and medullary tubule (D, *arrow*) epithelial cells, but not in glomerular epithelial cells. **(b)** The mean density of Na^+^-K^+^-ATPase staining in the kidneys of different groups is showed in the graph.

RDNX: renal denervation group; WKY: Wistar Kyoto rats; C: renal cortex; OSOM: outer stripe of the outer medulla; ISOM: inner stripe of the outer medulla. Significances (P < 0.05) are * vs. sham.

With positive expression as cytoplasm into brownish-yellow staining, the mean density of Na^+^-K^+^-ATPase staining in the kidneys of the experimental groups was illustrated as shown in Fig. 4. Immunohistochemical analysis using computer-assisted image analyzer system (IPP 6) showed that renal denervation significantly decreased expression of Na^+^-K^+^-ATPase proteins in the renal cortex, outer stripe and inner stripe of the outer medulla as compared to sham group (0.0956 ± 0.0144, 0.1310 ± 0.0175, 0.1693 ± 0.0136, respectively, P < 0.05), while RDNX group was not significantly different from WKY group (p > 0.05). Sham and WKY groups showed significant differences for the expression of Na^+^-K^+^-ATPase proteins in the renal cortex, outer stripe and inner stripe of the outer medulla (all P < 0.05).

## Discussion

In this study, we explored whether sympathetic nerve involve water and sodium balance by regulating EO secretion and kidney Na^+^-K^+^-ATPase activity in SHR. The main findings of our study are as follows: first, EO levels significantly reduced after decreasing renal sympathetic nerve activity in SHR kidneys. Second, renal denervations decreased Na^+^-K^+^-ATPase activity of kidney, the expression of Na^+^-K^+^-ATPaseα1 subunit mRNA of kidney and the expression of Na^+^-K^+^-ATPase proteins in the renal cortex, outer stripe and inner stripe of the outer medulla in SHR compared with the sham group. Third, we observed a positively correlation between NE content and Na^+^-K^+^-ATPase activity. However, the correlation analysis showed no statistical difference between Na^+^-K^+^-ATPase activity and EO levels in the kidney. EO has high intrinsic affinity and selectivity for the Na^+^-K^+^-ATPase by interacting with the cardenolide receptor on the Na^+^-K^+^-ATPase^20^. This result may be limited by the number of animals involved in the experiment or more complicated relationship between EO and Na^+^-K^+^-ATPase in the kidney. Taken together, these new results speculate that sympathetic nerve is involved in the regulation of Na^+^-K^+^-ATPase activity through reduction of hypothalamic EO secretion, thus playing an important role in the water and sodium balance.

EO can regulate the sodium pump on the membrane of central nervous system (CNS), heart, blood vessel wall and renal tubules, and EO functions as regulator of water and sodium metabolism and blood vessel wall tension, which indicates that EO is essential to the pathogenesis of hypertension^21^^,^ 22. Circulating EO is elevated and positively correlated with BP in a variety of hypertensive states, including essential hypertension and secondary hypertension, especially in the normal renin or low-renin essential hypertensive patients^23^. Moreover, in several hypertensive animal models, especially in rats genetically predisposed to hypertension such as SHR and Milan hypertensive rats, the levels of EO were increased not only peripherally but also centrally and in relatively high concentrations in the hypothalamus and pituitary^8^^,^ 21. Subsequent work implicated that BP returned to normal after administration of EO specific antagonist Canrenone or anti-ouabain antibody^24^^,^ 25. Bigazzi *et al*.^26^ collected data from 2,638 white adolescents, including BP, anthropometric measurements, genomic DNA extraction collecting from saliva and urine sample and renal function evaluation, finding BP values were associated with LSS (lanosterol synthase, rs2254524), a missense variant of EO-related gene. Our present study explored whether EO levels reduced after decreasing renal sympathetic hyperactivity in SHR kidneys.

EO elevated BP by inhibiting the reuptake of NE from the perivascular adrenergic nerve endings and increasing sympathetic nerve activity^27^. Vatta *et al*.^28^ have found that EO may increase NE availability in the synaptic gap by affecting the release and uptake of neurotransmitter in hypothalamus, and in turn enhance sympathetic nervous system activity. Raina *et al*.^29^ examined the effects of EO-induced vasoconstriction in endothelium of mesenteric arterial or renal interlobar arterial, establishing that ouabain-induced vasoconstriction dependent on increasing NE release from nerve endings in arterial wall, and a component of ouabain-mediated vasoconstriction was increased possibly via sympathetic activation. This has been confirmed in our previous studies that surgical renal denervation can reduce sodium retention while could attenuate sympathetic nervous system activity in CNS, kidney and peripherals in SHR^19^. The present findings suggest that renal denervation reduced peripheral and hypothalamic sympathetic activity of SHR while lowered EO in serum, kidney, and hypothalamus. Thus, the interaction between EO and sympathetic nervous system is crucial for the development of hypertension.

EO is closely associated with the metabolism of water and sodium in hypertension. EO-induced modulatory pathway may be the key roles resulting to the long-term elevation of BP in central and peripheral^17^. Paczula *et al*.^30^ reported that EO have a role in the adaptation to both sodium depletion and loading. EO concentration was higher in salt-sensitive group compared to salt resistant group and decrease after low salt diet, suggesting EO involve in the development of sodium induced, low renin, and salt-sensitive hypertension^31^. Manunta *et al*.^32^ conducted a cohort study of 301 hypertensive patients and found a significant increase in BP with elevated plasma EO levels, whereas the relationship between BP and renin activity (PRA) was reversed. Meanwhile, the fractional excretion of sodium (FENa) was negatively correlated with EO from the first to the third quartiles, although the pressure–natriuresis relationship was inversely related to PRA.

These findings indicated the mutually independent and compensatory relationship between RAAS and EO in water and sodium metabolism. Leenen *et al*.^33^ demonstrated that the tissue and plasma EO content were significantly higher in SHR than in WKY rats on a normal diet. The increase of EO in central and peripheral SHR was more obvious after long-term high-Na^+^ diet. In addition, our previous studies showed that RDN improved impaired sodium excretion metabolism, delayed glomerular damage and reduced renal vascular resistance in RDNX group compared with the Sham group^19^. The results of the present study demonstrates that RDN could reduce the content of EO in kidney, as well as serum and hypothalamus.

The hypothalamus and adrenal glands are the main sites of EO synthesis and secretion^34^. EO mainly distributed in the adrenal gland with the concentration about 100 times higher than in plasma^3^. EO content of plasma was significantly reduced after adrenalectomy in rats, suggesting that the adrenal gland may be the main source of EO^35^. Murrell *et al*.^36^ explored the pathway of EO biosynthesis in Milan hypertensive rats by applying bioinformatics combined with microarray analysis and gene interference technology, and found that the key enzyme gene of EO biosynthesis could be detected in hypothalamus and adrenal gland. Moreover, the two genes coding for P450 side chain cleavage enzyme and Δ5-3β-hydroxysteroid dehydrogenase/Δ5-Δ4-isomerase enzyme were remarkably upregulated in hypothalamus, but not in adrenal gland, compared with normotensive Milan rats. Our study found that RDN caused decrease of EO levels in the peripheral, renal, and hypothalamus of SHR. Notably, it had no effect on EO levels in adrenal tissue. We speculate that the synthesis and secretion of EO may have self-feedback regulation in adrenal, which need further investigation.

Na^+^-K^+^-ATPase pumped sodium ions outside and potassium ions inside living cells, converting chemical energy into work. EO existing in human circulation has high intrinsic affinity and selectivity for the Na^+^ pump by interacting with the cardenolide receptor on the Na^+^ pump^2^^,^ 20, suggesting that EO may play an important role in the regulation of cellular electrolyte homeostasis^20^^,^ 37. Specifically, Na^+^-K^+^-ATPase incorporates in the cell membrane, which obtains energy through ATP hydrolysis and transports ions depending on an inverse electrochemical gradient to regulate intracellular sodium-potassium homeostasis, cell membrane excitability and metabolism^38^. NE uptake in the presynaptic membrane of adrenergic nerve endings depends on Na^+^-K^+^-ATPase activity.

Our previous study showed that the renal sympathetic nerve may play an important role in the regulation of Na^+^-H^+^exchange (NHE) in the kidney^39^. Low EO concentrations can ascend Na^+^-K^+^-ATPase and NHE1 activity, as well as NHE1 is required for the ouabain-induced increase in BP^40^. Recent investigation suggested that the cooperative effects Na^+^-K^+^-ATPase and NHE1 activities on renal tubular was involved in endogenous ouabain-like factors stimulated hypertension^41^. Ferrandi *et al*.^42^ suggested that the increase of circulating EO level was related to the elevation of BP and the activity of Na^+^-K^+^-ATPase in renal tubules. The activity of Na^+^-K^+^ -ATPase was upregulated after oral administration of EO inhibitor (PST 2238) at the dose of 10 mg/kg/day for four weeks in rats with hypertension. After five days with EO inhibitor (PST 2238), the expression of Na^+^ -K^+^ -ATPase was increased in renal tubular epithelial cells.

A series of clinical trials had evidenced efficacy and safety of RDN to treat hypertension^43^^-^ 45. The significant decrease of BP following RDN had already been confirmed in the previous examination from our laboratory^19^. However, early trials of RDN showed conflicting results, and RDN is unsuitable for all patients with hypertension^46^. The lack of significant improvement in BP may be related to multiple possible causes, including the technical failure of RDN procedure, partial regrowth of renal nerves after renal denervation, variable patient drug compliance and patient selection criteria.

## Conclusions

Several experimental and clinical studies provide insights into the notion that EO and Na^+^-K^+^-ATPase play an important role in hypertension and related organ complications. The EO content and Na^+^-K^+^-ATPase activity were both reduced after treatment through RDN, indicating that the hypertensive patients may benefit from RDN. However, extensive clinical trials were required to solve existing problems including patient selection criteria and the indicators for evaluating RDN efficacy. So, RDN may be a viable option and a promising approach for hypertension in the future.
